# An e-mental health intervention to reduce depression symptoms in individuals with obesity: study protocol for the randomized, controlled, two-armed, confirmatory LightMood trial

**DOI:** 10.1186/s13063-024-07970-9

**Published:** 2024-02-28

**Authors:** Dilara Kocol, Sheila Geiger, Adam Schweda, Jil Beckord, Theresa Schadendorf, Christoph Jansen, Anita Robitzsch, Eva-Maria Skoda, Martin Teufel, Alexander Bäuerle

**Affiliations:** 1 Clinic for Psychosomatic Medicine and Psychotherapy, LVR-University Hospital Essen, University of Duisburg-Essen, Essen, Germany; 2 Center for Translational Neuro- and Behavioral Sciences (C-TNBS), University of Duisburg-Essen, Essen, Germany

**Keywords:** eHealth, Depression, Obesity, Mindfulness, CBT, Online-intervention, Randomized controlled trial

## Abstract

**Background:**

Patients with obesity often experience psychological distress, specifically depression symptoms. Due to various barriers, such as limitations of healthcare offers, digital interventions, for example medical apps, can provide a suitable approach to support affected people. In the envisaged prospective randomized controlled trial, we aim to examine the efficacy of the LightMood intervention. The LightMood intervention is a manualized and user-centered, digital intervention for patients with obesity, with a duration of 4 months, which contains elements of cognitive behavioral therapy and mindfulness-based and skills-based exercises. We expect the LightMood intervention to be superior to treatment as usual (TAU) in terms of reducing depression symptoms.

**Methods:**

The trial incorporates four distinct measurement time points: the baseline assessment, the post-treatment assessment, and 1- and 3-month follow-up assessments. Furthermore, we implemented in-treatment assessments for both groups. Participants will be randomized into two groups (LightMood intervention vs TAU). The aim is to include 128 participants (64 per group) in the study. Inclusion criteria are patients who are obese, at least 18 years old, with a private Internet access, and with adequate digital literacy and show depression symptoms (PHQ ≥ 10). Exclusion criteria are weekly outpatient individual psychotherapy, obesity surgery within the last year or planned within the next 7 months, no private Internet access, and the prescription of a new psychotropic drug within the last 2 weeks. The primary outcome is the post-assessment reduction in depression symptoms. Secondary outcomes will include the improvement in self-efficacy, quality of life, mindfulness, reduction in eating disorder symptoms, and body mass index (BMI). Furthermore, we expect a positive development of depression symptoms throughout the different time points (T1, T2, and T3) in patients with obesity.

**Discussion:**

LightMood is an evidence-based, efficient, low-threshold online intervention that aims to reduce depression symptoms in people with obesity. Online interventions could offer a promising alternative to conventional face-to-face therapy. The primary objective of the current study is to add essential insight into the feasibility, efficacy, effectiveness, and acceptance of e-mental health interventions for people with obesity and depression symptoms.

**Trial registration:**

German Clinical Trial Register (DRKS), DRKS00029219. Registered on May 19, 2023

**Supplementary Information:**

The online version contains supplementary material available at 10.1186/s13063-024-07970-9.

## Background

Overweight and obesity have become one of the most emerging health issues and prevalent chronic diseases worldwide [1], which are defined as abnormal or excessive fat accumulation that can cause several health issues. According to data of the OECD Health Policy Study, around 1.9 billion people 18 years and older were overweight, of which over 650 million were obese [1]. Hereby, the global prevalence of obese people has almost tripled between the years 1975 and 2016. Obesity itself as a chronical disease is associated with various physical illnesses, such as type 2 diabetes, cancer, arthritis [2], high blood pressure, cardiovascular risk factors, and mental health problems, especially depression [3–5].

The outcomes of a conducted meta-analysis detected a complex reciprocal pathway between depression and obesity [6], which underlies the co-existence of both diseases [7]. Besides that, a study, showed that more than 50% of obese patients suffered from depression or adjustment disorders [8].

Several guidelines emphasized the importance of dealing with the psychological aspect of obesity, such as improving self-esteem, body image, quality of life, and the treatment of depression [9]. This highlights the importance of effective treatments for people with overweight and obesity, which should not only focus on weight reduction but also on addressing the mental burden, particularly symptoms of depression.

Treating the high number of obese people [10] requires a high and long-term treatment effort that has financial consequences and leads to an increased burden on the health care system. The outcomes of face-to-face treatments in these contexts showed that there was deficient proof of the “scalability, generalisability and long-term sustainability” of treatments conducted [11]. These conditions have led to the establishment and inclusion of eHealth applications in the therapy and treatment of this group of patients [12]. Digital interventions such as medical apps could overcome the barriers obese people experience especially in taking advantage of psychotherapy.

Digital interventions address many difficulties in the existing community care, such as the physical immobility of patients, fear of stigmatization, and regional differences in healthcare or long waiting times for treatment [13]. Various studies show that eHealth approaches are of great benefit to the patient care and can reach comparable effects to traditional face-to-face treatments [14].

Next to the positive influences and advantages of eHealth approaches in the health care system and therapeutic settings, there are still some downsides of this approach. The most prevalent barriers to eHealth engagement refer to the older population, due to the fact that older adults lack self-efficacy, knowledge, support, functionality, and information provision about the benefits of eHealth approaches. However, compared to traditional face-to-face healthcare, eHealth approaches have several strengths in terms of affordability, usability, privacy, and security [15].

Recently developed eHealth interventions adequately addressed the abovementioned barriers and their influence on the users’ adherence in terms of using such interventions for a longer period. Hereby, the importance of user-centered design approaches became apparent [16]. User-centered design approaches emphasize the importance of combining and including actively the needs and preferences of the users and the industry, during the design process of such approaches. The outcomes of a study that focused on the acceptance of e-mental health interventions in patients with obesity showed overall a moderate acceptance of such interventions [17]. Limitations of the study that could explain the moderate acceptance of such interventions are that the study was exclusively web-based; hence, it was mandatory to have Internet access, which led to a sample of patients with younger age. On top of that, the sex distribution is not representative of the overall population, based on the fact that a large number of women participated. Further limitations are the varying number of patients in each obesity grade and the BMI bias.

Besides that, the outcomes of a systematic review showed that the needs and interests of patients were mostly involved at a very late stage of the development process of eHealth innovations. This is a reason for a deficit in the influence of the content and design decisions from the perspective of the users. Hence, it is of high relevance to identify, integrate, and center the needs and interests of the users at an early stage of the development process [18].

Based on the outcomes of previously conducted studies which focused on the efficacy of eHealth interventions to reduce depression symptoms in individuals with obesity, it became apparent that there is a limited amount of existing eHealth interventions and efficacy trials, targeting individuals with obesity and depression symptoms [19–21]. An increase in the efficacy of eHealth interventions was notable, when eHealth intervention targeted both mental and physical health simultaneously, mostly focused on weight loss as the primary target, through the inclusion of interactive strategies, where participants of the interventions receive several exercises per week, such as food diaries, breathing exercises, and sheets with questions, which they can fill in. On top of that, based on the outcomes of systematic reviews, it can be concluded that interventions, including cognitive behavioral therapy (CBT) modules or techniques, which integrate guided self-help approaches and digital tools, led to a high acceptance and efficacy in the reduction of depression symptoms. To conclude, eHealth interventions are efficient in reducing depression symptoms in obese adults in particular. Even though there is proven user acceptance for e-mental health interventions that patients with obesity affected by mental health disorders would benefit from e-mental health interventions, it is important to explore the specific needs of the patients in order to develop tailored interventions. Due to this lack, the adherence and acceptance to these interventions is low and there are no targeted and individualized eHealth intervention to reduce depression symptoms in obese individuals.

Therefore, the LightMood e-mental health intervention was designed based on previous research and outcomes regarding the acceptance of e-mental health interventions and the needs and demands in terms of design and content of such interventions [17, 22]. The LightMood intervention is based on established and effective psychotherapeutic intervention techniques of Mindfulness-Based Stress Reduction (MBSR), CBT, and Acceptance and Commitment Therapy (ACT), which aims to reduce depression symptoms. Based on the previous study outcomes and the current body of research, we propose to conduct the randomized, controlled, two-armed, confirmatory intervention LightMood trial.

### Objectives and hypotheses

The objective of the proposed trial is to address depression symptoms in patients with obesity and provide a low-threshold e-mental health intervention. We aim to assess the efficacy of the e-mental health intervention LightMood in patients with obesity and depression symptoms.

The primary hypothesis is that we expect the LightMood intervention to be superior to TAU in terms of reducing depression symptoms (T1) in patients with obesity.

Secondary hypotheses are that we expect the LightMood intervention to be superior to TAU in terms of improving self-efficacy, quality of life, mindfulness, reducing eating disorder symptoms, and reducing body mass index (BMI). Furthermore, we expect a positive development of depression symptoms throughout the different time points (T1, T2, and T3) in patients with obesity. All in all, it is expected to reach improvement by T1 and sustained gains at T2 and T3.

Other study goals are to evaluate client satisfaction, usability, and adherence of and to the LightMood intervention. Furthermore, we will assess usage behavior and explore relations between the observed usage behavior and other study outcomes.

## Methods

This study protocol is reported according to the Standard Protocol Items: Recommendations for Interventional Trials checklist (see online supplemental material, [23]). In case of important protocol modification, it will be reported to the ethics committee of the Medical Faculty, University of Duisburg-Essen, and the trial registration will be updated.

### Study design

The study is an online-based study, which is coordinated in the clinic for psychosomatic medicine and psychotherapy, LVR-University Hospital Essen, University of Duisburg-Essen in Essen, Germany. The study comprises a prospective, randomized controlled trial with two parallel arms. The proposed trial incorporates four distinct measurement time points: the baseline assessment (T0) before randomization, a post-treatment assessment (T1), and a 1-month and 3-month follow-up assessment (T2 and T3). Additionally, continuous assessments are planned during the experimental and control intervention (in-treatment assessment); Table 1 gives an overview of the assessment schedule.

**Table 1:** Overview of the assessment schedule

Measures	Baseline	In-treatment assessment	End of treatment	Follow-up assessments	Drop-out assessment
**Primary outcome**					
CESD-R	x		x	x	x
**Secondary outcomes**					
GSES	x		x	x	
WHOQOL	x		x	x	
FFA	x		x	x	
EDI-2	x		x	x	
EDE-Q8	x		x	x	
CSQ-I			x		x
SUS			x		x
Usage behavior		x			
UTAUT	x				
PHQ-4	x	x	x	x	x
DT	x	x	x	x	x
BMI	x		x	x	
Sociodemographics, medical characteristics	x				

*CESD-R* Center of Epidemiologic Studies Depression Scale-Revised, *GSES* General Self-Efficacy Scale, *WHOQOL* World Health Organization Quality of Life, *FFA* Freiburger Fragebogen zur Achtsamkeit,*EDI-2* Eating Disorder Inventory Bulimia subscales, *EDE-Q8* Eating Disorder Examination Questionnaire, measuring Eating Disorder specific Symptoms, *CSQ-I* Client Satisfaction Questionnaire Internet-based, *SUS* System Usability Scale, *UTAUT* Unified Theory of Acceptance and Use of Technology questionnaire, *PHQ-4* Patient Health Questionnaire-4, *DT* distress thermometer, *BMI* body mass index

A participant who has decided to quit during the intervention counts as a dropout (no login for 6 weeks is considered as a dropout). Patient who drop out of the study will be asked to complete a dropout assessment. The participant who decides to drop out will be contacted (if written permission was given) by study personnel so that the drop-out assessment can be sent to the participant.

### Participant eligibility and recruitment

Participants will be included if they have a BMI ≥ 30, which is the cut-off score for obesity, an age of 18 and higher, high perceived depression symptom (PHQ-8 ≥ 10), adequate digital literacy, and Internet-enabled device. Furthermore, giving informed consent is mandatory. The intervention will be conducted in the German language.

Participants who had a bariatric surgery within the past year or planned within the next 7 months, have weekly individual psychotherapy, have no private Internet access, and had a regulation of a new psychotropic drug within the last 2 weeks will be excluded. Apart from that, there are no prohibitions regarding the utilisation of health services.

Participants are going to be recruited by study personnel. Flyers and posters will be spread in specialized clinics. In addition, information about the study and the involved staff will be presented online via social media (e.g. Facebook) and the study website. If participants are interested, they can use the link or QR code to go to the study website. Interested participants are able to ask questions regarding the study via email and phone and in person.

#### Intervention

Eligible patients will be randomly assigned to either the experimental intervention or the control condition (TAU). Both interventions last 4 months. No adaption or modification of the intervention is planned in any case.

#### Experimental intervention

The LightMood intervention is an evidence-based developed, manualized, user-centered, digital intervention for obese patients to reduce depression symptoms [17, 22]. Furthermore, the intervention is based on established, effective psychotherapeutic intervention techniques of MBSR, CBT, and ACT used during the treatment of people with obesity. It aims to improve depression symptoms management, mental strength, psychological resources, self-efficacy, and enhancement of physical activities. This intervention is designed to accompany participants with obesity and depression symptoms for 4 months. The LightMood intervention consists of total of 16 modules. Hereby, the participants are provided in total with 8 educative modules and 8 interactive modules, called trainings (see Table 2). Every module lasts approximately 30 min. The 8 trainings involve various exercises, an individual skills box, audio-guided mindfulness videos, and diet and physical exercise plans. Hence, during each module, patients collect helpful skills in their individual skills boxes. The intervention also involves homework assignments and MBSR trainings that should be integrated into the daily routines of the participants. Each LightMood training is conceptualized to be completed within 1 week; after the training week, the next module is delivered. The participants must complete prior modules to receive access to the next one. The participants do not lose access to prior modules, when the next module is delivered. The modules which integrate a diary function or making a plan are possible to be used more than once. A module is considered to be completed, when the participant goes through every page of the exercise and conducts the exercise, by for example filling in the assignments. When a new module and training is available, participants receive a motivational, supportive, and encouraging notification via email. To ensure intervention adherence and fidelity, all participants in the experimental intervention group will receive notifications that inform them when a new module is available. All notifications and reminders are standardized. The training can be conducted on the participants’ private PC, tablet, or smartphone.

**Table 2:** Overview of the LightMood interventional

Module	Educative modules	Training
Technical introduction and mindfulness	Quick introduction, structure of the LightMood intervention, introduction to mindfulness	Mindfulness exercises
Mental health	Education about mental health, influence on one’s physical health	Exercise against brooding, creating a list with activities
Emotions	Accepting and dealing with emotions such as depression, influence on eating behavior	Strategies to control negative emotions
Self-efficacy	Importance of self-efficacy, need to integrate activities or sources in life that enhance self-efficacy	Exploring areas in life that enhance self-efficacy
Health-related behavior	Different aspects of promoting physical and mental health during obesity (diet, creating daily structure)	Setting SMART goals, focusing on health-promoting behavior
Stress management	Education about the impact of stress on daily life, nutrition, mental health, etc.	Discovering specific trigger of stress and stress-related behavior, goal of changing habits and reducing stress in the long term
Exercise and relaxation	Finding a healthy balance between exercise and relaxation	Creating an individual plan with exercises and breathing exercises
Positivity and outro	Making room for positive experiences; noticing positive accomplishments. Review of LightMood intervention	Determining energy-giving and energy-taking factors, focusing on those that give more positive energy

#### Control intervention

In the proposed RCT, the intervention group will be compared with a control group. The intervention will be compared to the usual care the participants receive in conventional healthcare in Germany. Hence, there is no restriction regarding access to therapeutic and/or medical services during the trial. Besides that, the participants in the control condition do not receive any additional treatment. The control group participants will not have access to any content of the LightMood intervention. Instead, they will receive every week notifications via mail for the in-treatment assessment, which will be conducted online by the participants.

#### Outcomes

The primary outcome is the reduction in depression symptoms at the end of treatment (T1; see Table 1). To measure *depression symptoms*, we will use the German Version of the Center of Epidemiologic Studies Depression Scale—Revised [24]. The CESD-R is a valid measurement instrument, used to record the diagnosis and severity of depression disorders according to DSM-5 in clinical studies [25, 26]. The CESD-R consists of 20 items covering nine different symptom areas [24].

Secondary endpoints for this study include (1) self-efficacy, (2) quality of life, (3) mindfulness, (4) eating disorder symptoms, (5) BMI, (6) treatment satisfaction, (7) usability, (8) usage behavior, (9) predictors of usage behavior, and (10) course/development of depression symptoms. All scales used are presented in German.

1. *Self-efficacy*: for the assessment of self-efficacy, we will use the German version of the General Self-Efficacy Scale [27]. The GSES is a self-report measure of self-efficacy and optimistic self-beliefs related to coping with a variety of difficult demands in life as well as coping with all kinds of stressful life events. Responses are rated on a 4-point Likert scale for all 10 items.
2. *Quality of life*: The World Health Organization Quality Of Life Questionnaire [28] is used to assess quality of life. The WHOQOL assesses the individual’s perception of their own position in life in the context of the culture and value systems in which the person lives and in relation to their goals, expectations, norms, and concerns. The WHOQOL measures life satisfaction and self-esteem using 26 items. Items are rated on a 5-point Likert scale from 1 (low satisfaction) to 5 (high satisfaction).
3. *Mindfulness:* to assess mindfulness, we will use the validated German version of the Freiburg Mindfulness Inventory [29]. It consists of 14 items, and all responses are given on a 4-point Likert scale.
4. *Eating disorder symptoms:* The Bulimia Subscale of the Eating Disorder Inventory-2 [30] and the Eight-Item Short Version of the Eating Disorder Examination Questionnaire [31] are used to record eating disorder-specific symptoms. The EDI-2 bulimia subscale consists of 7 items measuring symptoms of bulimia, with a focus on binge eating [30]. Items are measured on a 6-point Likert scale ranging from 0 (never) to 6 (always). The total score ranges from 0 to 42. The EDE-Q8 is also used to assess the psychopathology of eating disorders. The EDE-Q8 contains two items each on the subscales restraint, concern about eating, concern about weight, and concern about body shape. The EDE-Q8 consists of 5 items measuring the psychopathology of the eating disorder over the past 28 days on a 7-point Likert scale from 0 (no day) to 6 (every day), and 3 items measuring occurrence and assess the frequency of core eating disorder behaviors on a scale from 0 (never) to 6 (every time) [31].
5. *BMI*: Body mass index is a measure of an individual’s body weight in relation to their height. Height and weight are recorded via self-reports [32].
6. *Treatment satisfaction:* The Client Satisfaction Questionnaire [33], adapted to Internet-based interventions, is used to record treatment satisfaction. The CSQ-I consists of 8 items, which are answered with a 4-point Likert scale. The CSQ-I is a measure for considering user satisfaction in the overall evaluation of eHealth interventions.
7. *Usability:* The System Usability Scale [34] is used to measure usability. The SUS consists of 10 items, which are answered with a 5-point Likert scale. The SUS is a measure for evaluating the usability of a system.
8. *Usage behavior:* The time to dropout and the usage behavior are calculated based on the number and type of modules completed, the time per day, the type and number of modules started, the time since the last login, the frequency of login, the frequency of each module, the time spent on each module, the percentage of each module completed, the type and number of videos and audios started, and the type and number of videos and audios finished. The data is assessed via the backend of the intervention.
9. *Predictors of usage behavior:* To measure predictors of usage behavior, the validated Unified Theory of Acceptance and Use of Technology questionnaire [35] is used. The UTAUT evaluates health-related Internet use, the acceptance of eHealth measures, and the barriers and resources of eHealth use.
10. *Course/development of depression symptoms and distress*: Depression and anxiety symptoms: The Patient Health Questionnaire-4 [36] in its validated German version is used to measure depression and anxiety symptoms. The PHQ-4 is a proven short questionnaire consisting of four items: two items on depression symptoms (PHQ-2) and two items on generalized anxiety symptoms (Generalized Anxiety Disorder Scale-2 (GAD-2)). Responses are rated on a 4-point Likert scale for all four items. The PHQ-4 will be applied to all patients throughout the study.
11. *Distress:* Distress and its progression, measured with the Distress Thermometer [37], will be analyzed in all patients throughout the study. The DT is an efficient and convenient validated measurement tool for assessing exposure. It is an established and rapid screening tool for stress, acting as a visual thermometer on a scale of 0 (no stress) to 10 (more extreme stress).

#### Trial procedure and timeline

**Fig. 1:**
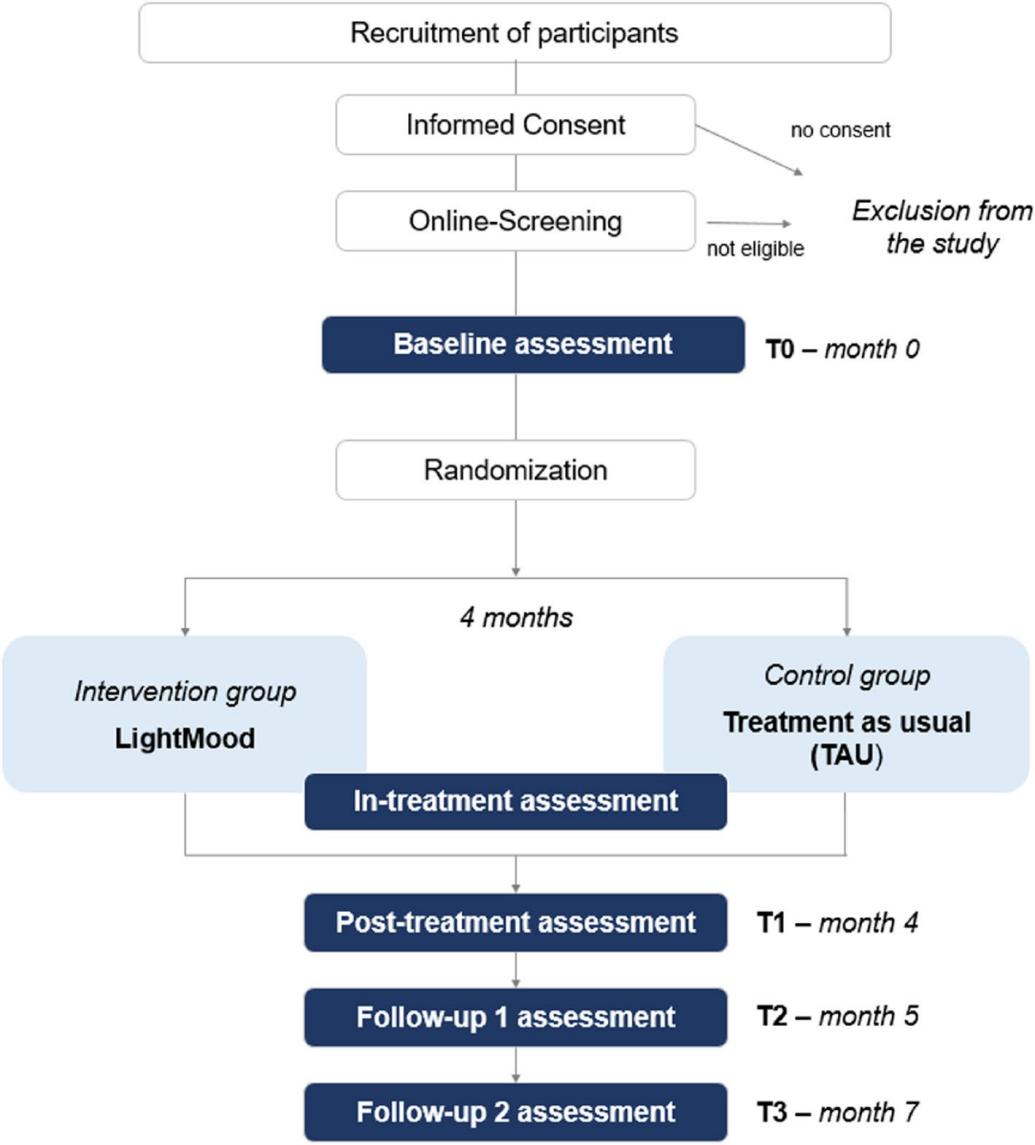
Trial flow of the LightMood trial

An overview of the trial flow and assessment schedule is presented in Fig. 1 and Table 1, respectively. Trial duration for participants comprises 7 months (including 4 months of intervention and 3 months of follow-up period).

Once the interested participant contacts the study staff, a screening for eligibility will be applied and the patient will receive an explanation about the study conditions, data storage, and data safety. The patient will be included in the study, if inclusion criteria are met and exclusion criteria do not apply and the participant has given written informed consent. Informed consent will be obtained by trained and supervised study personnel. Once the participant is included in the study and the baseline assessment is completed, allocation into one of the following study groups will be applied via randomization: LightMood intervention (experimental group) or TAU (control group). The patients will be informed about their group allocation result and will get access to the LightMood intervention (only the intervention group), which takes 4 months in total. After completing the 4-month intervention, participants receive the post-treatment assessment (intervention group and control group). Furthermore, there is a 1- and 3-month follow-up assessment.

Standardized notifications and reminders are sent to patients to increase the completion rate and reduce the loss to follow-up rate: a notification is sent when an assessment is due and a reminder message is sent 1 week and 2 weeks after the scheduled time of the assessment.

#### Sample size calculation

The calculation of the sample size was performed using the R-package “Superpower” [38]. Our primary hypothesis is that the intervention will be superior in terms of reducing depression symptoms compared to TAU (control group) at T1. To assess the efficacy of the intervention, we will conduct an ANCOVA analysis with the primary outcome variable at T1 as the dependent variable, the group variable (intervention vs. control group) as a between-subject factor, and the individual baseline levels at T0 as a covariate. Subsequently, a mixed ANOVA analysis with the respective measurements (T0–T3) as a within-subject factor and the group variable as a between-subject factor will be used to explore the intervention’s effect at follow-up.

For the sample size calculation, we selected a preferred test power of 1 − *ß* = 0.80. We anticipate a moderate effect size of *d* = 0.5 for the baseline-adjusted comparison between the intervention and control group at T1. Based on previous studies, we expect the correlation between repeated measures to be *r* = 0.5 [39].

According to the power analyses, a total effective sample size of *n* = 98 (49 participants per group) will provide a statistical power of 80.34 for the ANCOVA analysis mentioned above. With the same number of participants, we achieve a power of 95.8 for the mixed ANOVA analysis mentioned earlier, assuming a conservative assumption that the sole difference between the intervention and control group occurs at T1.

Considering a dropout rate of 30% from T0 to T3, based on existing literature on eHealth trials [40], we estimate that 128 participants (64 per group) will be required. This dropout rate includes loss to follow-up.

#### Randomization, blinding, and allocation concealment mechanism

The study will utilize randomization (specifically, 1:1 block randomization, with 20 participants per block). Randomization will be applied through a standard computer algorithm (automatically after patients complete the baseline assessment) with no engagement of study personal. The allocation sequence was generated by a study-independent data and information technology manager (author CJ) via a standard computer algorithm. Further study personnel is not informed about the allocation sequence. Stratification will not be applied, as it is anticipated that the randomization process will achieve a balance of prognostic factors. Upon enrollment and completion of the randomization process, the participants will be informed via an automatically generated e-mail of their group allocation. In case of any enquiries, the patients are able to contact the study team via phone or e-mail.

Due to the nature of the trial, blinding the patients will not be feasible. However, to ensure unbiased analyses, the assessors and statisticians involved in the primary analysis will be kept blinded to group allocation. There are no intentions to unblind the assessors or statisticians.

The applied central randomization approach (via a standard computer algorithm) as well as the automatic communication (via the computer system) of the group allocation to the patients will ensure concealed allocation.

#### Data management, data storage, and dissemination policy

In order to protect the confidentiality of participants, their data will be pseudonymize. In accordance to the Good Clinical Practice guidelines, important trial data will be archived for 10 years after the trial’s completion or termination [41]. Patient documents will be stored in compliance with hospital regulations, and access to stored data during and after the trial will be restricted to authorized staff only.

Once the major results of the trial are published, the collected data will be made available in an anonymous format upon reasonable request. Additionally, we will store and provide access to the statistical analysis plan and other relevant documents upon request. Access to the data storage will be limited to authorized personnel exclusively. Patient consent forms will include a section addressing the aforementioned aspects of data storage and sharing.

To disseminate the progress and outcomes of the trial, we have devised several measures. Firstly, we will establish a project homepage that will offer regular updates to the public on the trial’s development, opportunities for participation such as workshops and conferences, and significant findings. This website will remain active even after recruitment is closed, serving as a central hub for future research and dissemination of results. The main trial results will be published in an open-access, peer-reviewed journal and made publicly available through the clinical trial registry. Furthermore, we will present the findings at conferences and communicate scientific results in plain language through press releases, social media, and patient forums. To ensure the results reach the affected population and the general public, we will collaborate with patient representatives and organizations to develop targeted, patient-oriented information campaigns.

#### Statistical methods

The primary objective of this trial is to evaluate the efficacy of the intervention in terms of reducing depression symptoms after the intervention, compared to the control group. To achieve this, we will employ an analysis of covariance (ANCOVA) with the outcome measured at T1, using the between-subject factor of the group (intervention vs. control) and the participants’ baseline values (T0) as a covariate. The primary analysis will be conducted on the intention-to-treat population, with missing data imputed in cases of dropout. Imputation will be performed using the SPSS multiple imputation module, employing “monotone missing pattern” and incorporating complete data for sex, age, and baseline measurements of primary and secondary outcomes. The imputation process will be executed with 3000 imputations, using the analysis date as the seed. Interim analyses are not planned.

Subsequent mixed ANOVAs will be conducted with a between-subject factor representing group membership (intervention vs. control) and a within-subject factor corresponding to the respective measurement points (T0–T3). These analyses will assess whether the effects of the intervention are sustained during the follow-up period. Similar approaches will be applied for the analysis of additional clinical endpoints. In the event of substantial deviations from normality or homoscedasticity assumptions, non-parametric tests, robust procedures such as robust regression analyses, generalized estimating equations, or robust mixed linear models will be employed instead. Corrections, such as Huynh-Feldt or Greenhouse-Geisser, will be implemented if the assumption of sphericity is violated in the mixed ANOVA.

Descriptive analyses will be conducted to assess treatment satisfaction, usability, and usage behavior. For comparisons of demographic and baseline parameters between the two groups, *t*-tests (or Mann-Whitney *U*-tests in the case of non-normality) and *χ* ^2^ tests (or Fisher’s exact tests for variables with small cell sizes) will be utilized. Further mixed ANOVAs will be used to assess the impact of the intervention on the secondary outcome measures, such as eating behaviors (see Table 1). Also here, we will resort to more robust models in case of a violation of assumptions. Also, in case of a significant effect of our intervention on the primary outcome, we will assess whether this result is mediated by increases or decreases in scores concerning eating behavior. Here, we will use mediation analyses on mixed linear models or robust alternatives (such as mixed linear models with bootstrapping). We will not perform subgroup analyses. Additionally, analyses of variance and *χ* ^2^ tests will be employed to examine potential differences in sociodemographic and clinical data among various subgroups, such as dropouts, participants who completed the intervention, and those who benefitted from the intervention.

Exploratory analyses, including regression analyses, will be conducted to investigate the relationship between usage behavior and clinical outcomes, considering all assessments including in-treatment assessments. Secondary endpoints will include the examination of time to dropout using the Kaplan-Meier estimate and Cox proportional hazards regression. Here, a standard proportional hazards model—using the *coxph* function from the *survival* package in R—will be applied. Differences in time to dropout between the treatment and the control group will be assessed. Also, the baseline values of the relevant psychometric measures of depression and mental distress (CESD-R, PHQ-4, DT), self-efficacy (GSES), quality of life (WHOQOL), and eating behavior (EDI-2, EDE-Q8), as well as sociodemographic and medical characteristics (gender, SES, BMI), will be entered into the regression model to explore potential determinants of dropout. In case of large deviations from constant hazards (which will be tested using the *cox.zph* function from the abovementioned package), we will explore underlying reasons and might, for instance, further assess non-linear effects or models with stratification according to certain covariates.

To ensure safety assessment, separate tabulations and line listings of adverse events and severe adverse events will be generated for comprehensive analysis.

#### Patient and public involvement

Patient engagement was a key aspect in the development of the intervention and the planning of the proposed trial. The intervention itself was designed using a user-centered design approach [22]. Additionally, we have conducted assessments to evaluate the acceptance of such an e-mental health intervention and identify potential drivers and barriers to its usage [17].

Patient representatives actively participated in creating patient-friendly summaries and consent forms. Furthermore, we will maintain close collaboration with patient representatives and self-help organizations to ensure effective dissemination of the trial results within the target group. To enhance accessibility, the comprehensibility, illustration, and processing duration of the final case report forms were tested.

To keep the public informed, the trial results will be published in scientific journals and presented at professional congresses. Moreover, the findings will be shared in easy-to-understand language on social media channels and the study website. This approach aims to make the results accessible and understandable to a wider audience.

## Discussion

In our prospective, randomized controlled LightMood trial with two parallel arms, we aim to assess the efficacy of a manualized, user-centered, e-mental health intervention for participants with obesity to reduce depression symptoms. The aim is to offer an evidence-based, efficient, low-threshold online intervention.

The provision of care via digital approaches aims to keep costs low for treating staff and premises, to offer support to many people at the same time, to promote continuity of care by being independent of time and place, and to overcome the insufficient availability of specialized treatments by improving outreach. Thus, the intervention has socioeconomic and health-economic benefits. By evaluating the efficacy of the LightMood intervention, relevant findings for e-mental health interventions in general and in the context of obesity and mental health burden could be derived. Accordingly, other patient groups could benefit from similar e-mental health approaches as well. Exploring relations between actual usage behavior and mental health outcomes could be of particular interest to eHealth research in general.

Several guidelines emphasized the importance of dealing with the psychological aspect of obesity, such as improving self-esteem, body image, quality of life, and the treatment of depression [9]. The initial results of e-mental health interventions have been promising for people with obesity [19–21]. Accordingly, RCTs are needed to evaluate e-mental health interventions in obese. Given the increasing impact of digital technologies in daily life, the online LightMood intervention has the potential to reach a high number of people with obesity and to overcome barriers that patients face in analog fact-to-face care (e.g., long waiting times for psychotherapy).

Due to its accessibility everywhere at any time, the LightMood intervention enables people with obesity and mental health burden to participate in an online intervention regardless of barriers in the current healthcare system.

However, the trial has some limitations. The study only includes participants with access to the Internet, as access is required in the intervention. Furthermore, blinding to group allocation of the patients is not possible due to the nature of the present trial. However, this is a common issue in digital health trials. Assessors and statisticians will be blinded.

In conclusion, this intervention is addressing a large treatment and research gap, based on the fact that there are very few digital interventions targeting this population. On top of that, this intervention has the strengths of their user-centered approach and combined treatment of depression and obesity.

## Trial status

The LightMood trial is not yet recruiting as of the date of the publication. The planned date of recruiting is the first of August 2023. Recruitment is expected to be completed in May 2024. This trial has been approved by The Ethics Committee of the Medical Faculty of the University Duisburg-Essen (protocol version #1, dated July 2023, drks.de #22-10882-BO).

### Supplementary information

**Additional file 1. **SPIRIT checklist.

## Data Availability

In accordance to the Good Clinical Practice guidelines, important trial data will be archived for 10 years after the trial’s completion or termination. Patient documents will be stored in compliance with hospital regulations, and access to stored data during and after the trial will be restricted to authorized staff only. Once the major results of the trial are published, the collected data will be made available in an anonymous format upon reasonable request. Additionally, we will store and provide access to the statistical analysis plan and other relevant documents upon request. Access to the data storage will be limited to authorized personnel exclusively.

## Abbreviations

CBT: Cognitive behavioral therapy
TAU: Treatment as usual
MBSR: Mindfulness-based stress reduction
ACT: Acceptance and commitment therapy
CESD-R: Center of epidemiologic studies depression scale-revised
GSES: General self-efficacy scale
WHOQOL: World Health Organization quality of life
FFA: Freiburger Fragebogen zur Achtsamkeit
EDI-2: Eating Disorder Inventory Bulimia subscales
EDE-Q8: Eating disorder examination questionnaire, measuring eating disorder specific symptoms
CSQ-I: Client satisfaction questionnaire internet-based
SUS: System usability scale
UTAUT: Unified theory of acceptance and use of technology questionnaire
PHQ-4: Patient health questionnaire-4
DT: Distress thermometer
BMI: Body mass index
